# Understanding Interleukin 33 and Its Roles in Eosinophil Development

**DOI:** 10.3389/fmed.2017.00051

**Published:** 2017-05-02

**Authors:** Laura K. Johnston, Paul J. Bryce

**Affiliations:** ^1^Department of Medicine, Division of Allergy-Immunology, Northwestern University Feinberg School of Medicine, Chicago, IL, USA

**Keywords:** interleukin 33, eosinophils, stem cells, ST2/ST2L, asthma, allergy and immunology

## Abstract

Over the last decade, significant interest in the contribution of three “epithelial-derived cytokines,” such as thymic stromal lymphopoietin, interleukin 25, and interleukin 33 (IL-33), has developed. These cytokines have been strongly linked to the early events that occur during allergen exposures and how they contribute to the subsequent type 2 immune response. Of these three cytokines, IL-33 has proven particularly interesting because of the strong associations found between both it and its receptor, ST2, in several genome-wide association studies of allergic diseases. Further work has demonstrated clear mechanisms through which this cytokine might orchestrate allergic inflammation, including activation of several key effector cells that possess high ST2 levels, including mast cells, basophils, innate lymphoid cells, and eosinophils. Despite this, controversies surrounding IL-33 seem to suggest the biology of this cytokine might not be as simple as current dogmas suggest including: the relevant cellular sources of IL-33, with significant evidence for inducible expression in some hematopoietic cells; the mechanistic contributions of nuclear localization vs secretion; secretion and processing mechanisms; and the biological consequences of IL-33 exposure on different cell types. In this review, we will address the evidence for IL-33 and ST2 regulation over eosinophils and how this may contribute to allergic diseases. In particular, we focus on the accumulating evidence for a role of IL-33 in regulating hematopoiesis and how this relates to eosinophils as well as how this may provide new concepts for how the progression of allergy is regulated.

## Introduction

Allergic diseases are increasing worldwide, and the mechanisms of both allergic sensitization and the subsequent effector responses following reexposure, including by eosinophils, are still not fully understood. Significant focus has recently centered on three cytokines as regulating type 2 immunity in allergic individuals: thymic stromal lymphopoietin (TSLP), interleukin 25, and interleukin 33 (IL-33). Evidence shows that these cytokines influence allergic mechanisms that include activating type 2 innate lymphoid cells (ILC2s), the development of T helper type 2 (Th2) cells, and several other effector cells, including eosinophils. Development of antibodies targeting these epithelial-derived cytokines in allergic disease is underway: an antibody against TSLP is currently showing beneficial effects in patients (1), and anti-IL-33 has entered phase two in clinical trials (2). IL-33 in particular seems important for eosinophil biology, both in homeostatic development and activation during disease. As an example, a recent loss-of-function mutation in *Il33* was identified in patients and associated with reduced blood eosinophils and protection from asthma (3). In this review, we will discuss not only how IL-33 contributes to eosinophil biology but also recent evidence for roles of IL-33 in eosinophil development, which challenge the accepted view of IL-33 as regulating mainly local tissue responses.

## Controversies in IL-33 Biology

Interleukin 33 was originally found as a nuclear factor of high endothelial venules and termed NF-HEV (4). Interest was reignited when computational predictions showed a characteristic β-trefoil domain similar to the IL-1 family of cytokines (i.e., IL-1α, IL-1β, and IL-18), thus becoming the 11th family member known as IL-33 (5). Notably, Schmitz et al. also identified IL-33 as the ligand for the previously orphan receptor suppression of tumorigenicity 2 (T1/ST2) (also called interleukin 1 receptor-like 1), which had already been associated with allergic disease; indeed, IL-33 injection into mice led to increased spleen weight, IgE, type 2 cytokines, mucus production by epithelial cells, and significant eosinophilia. Thus, much of the early research on the IL-33/ST2 axis defined clear roles in type 2 immune-mediated responses.

Despite this, controversy has surrounded IL-33 with unanswered questions related to its cellular sources, subcellular location, and release mechanisms. While many have assigned IL-33 as an epithelial-derived cytokine, we and others have established that immune cells also express IL-33 upon activation, including macrophages, dendritic cells (DCs), eosinophils, B cells, monocytes, and mast cells (6–11). While IL-33 has been shown to be present in surface epithelial cells from human biopsies (12), studies of gene expression using a reporter mouse demonstrate that type 2 pneumocytes are the dominant cell expressing IL-33 within the lung (6) and that Clara cells, ciliated epithelial cells of the bronchiolar system, do not express IL-33 unless inflammation is induced (13). A point of contention is the question of functional contributions of structural- vs immune cell-derived IL-33. In mouse studies addressing this question, IL-33 from macrophages (14), DCs (8), and monocytes (7) are sufficient to support the development of Th2 responses and eosinophilia. In contrast, one study showed that transferring IL-33 knockout (KO) bone marrow into irradiated mice had no effect on allergic inflammation (15). Further studies are needed, especially given the significant caveat that several of these immune cells are highly radiation resistant. Taken together, while current evidence shows clear roles for immune cell-derived IL-33, the relative importance of structural- vs immune cell-derived IL-33 remains to be determined.

The mechanism of how cells release IL-33 is also subject to debate. IL-33 has been described as residing exclusively in the nucleus of structural cells (16), yet evidence now suggests this conclusion is likely influenced by alterations in the IL-33 protein upon fusion with fluorescent tags used to track the protein; a more careful assessment of native IL-33 revealed both nuclear and cytoplasmic presence (17) in endothelial cells and fibroblasts—indeed, we demonstrated cytoplasmic location within mast cells (18). Unlike many other IL-1 family members, IL-33 does not utilize the inflammasome pathway (19). Although its release upon necrotic cell death gave rise to the concept of IL-33 as an “alarmin” (20), mechanical stress could also induce IL-33 release from fibroblasts in the absence of necrosis (17). Relevant to allergy, IL-33 release was shown in response to the established adjuvant aluminum hydroxide (alum) (21). Allergens interact with mucosal tissue surfaces in many ways including through toll-like receptors 2 and 4 (TLR2 and TLR4), dectin-1, and protease-activated receptor-2 (PAR-2) (22), wherein dectin-1 and PAR-2 are necessary for allergen-induced increases in IL-33 in lung tissues (23, 24). The source of such increases in IL-33 remains poorly defined: while several studies have described IL-33 secretion from structural cells (17, 25–27), mast cells (28), DCs (9), and human monocytes (29) can also express and release IL-33. Since alveolar macrophages serve as a front line of allergen exposure in the airway and TLR ligands being shown to stimulate IL-33 in macrophage (30), immune cells as a source of IL-33 might occur both within the airspace and tissues.

## The Effects of IL-33 on Mature and Developing Eosinophils

Eosinophil expansion is a hallmark of most allergic disease, but the underlying mechanisms are not fully understood. Several scenarios may explain this expansion: proliferation of resident eosinophils, increased trafficking of blood eosinophils into tissues, increased output from the bone marrow, increased survival, and local maturation of progenitors in tissues. Mature eosinophils, which respond to IL-33, do not seem to possess a robust proliferative capacity, and so focus has been on developmental processes. The current knowledge of the ways in which IL-33 influences eosinophil biology during homeostasis and disease is discussed below.

### IL-33 on Mature Eosinophils

**Figure 1:**
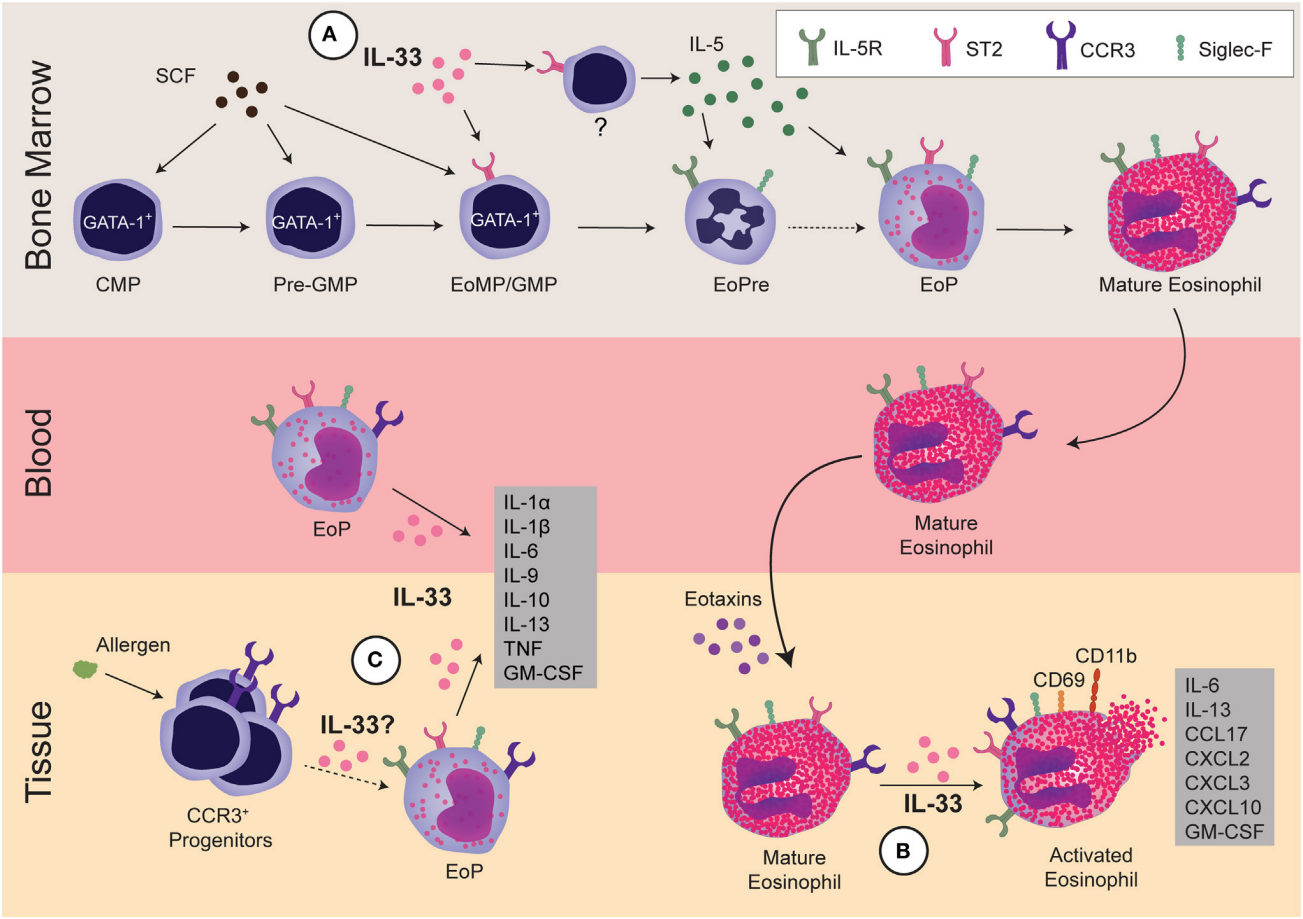
**Regulation of eosinophils by interleukin 33 (IL-33)**. IL-33 regulates eosinophils during three stages: development within the bone marrow, activation of mature cells, and development and/or activation of progenitors within the tissue. **(A)** In the bone marrow, GATA-1^+^ common myeloid progenitor (CMP) differentiates into GATA-1^+^-pre-granulocyte macrophage progenitor (Pre-GMP) (Lin^−^Sca-1^−^c-kit^+^CD41^−^CD16/32^−^CD105^−^CD150^−^GATA-1^+^), then to GATA-1^+^-granulocyte and macrophage progenitor (GMP), also known as eosinophil/mast cell progenitors (EoMP, Lin^−^Sca-1^−^c-kit^+^CD41^+^CD16/32^+^GATA-1^+^). At this early stage, IL-33 regulates the expansion of eosinophil precursor (EoPre) through differentiation of the EoMP/GMP. Since IL-33 also upregulates IL-5Rα on EoPre, it regulates commitment to the eosinophil lineage. Simultaneously, IL-33 induces another currently unidentified cell within the bone marrow to make IL-5, which promotes final eosinophil maturation. **(B)** In the tissue, IL-33 can activate mature eosinophils, leading to cytokine production and upregulation of CCR3, CD69, and CD11b. Notably, IL-33-driven production of GM-CSF and IL-13 promote eosinophil survival and differentiation of alternatively activated macrophages, respectively. **(C)** Finally, IL-33 can regulate EoP outside of the bone marrow. IL-33 increases the number of EoP in blood as well as activates EoP to produce many cytokines. Although allergens increase CCR3^+^ progenitors in tissue, it is unclear if increases in EoP in asthma patients are due to EoP leaving the bone marrow or extramedullary eosinophilopoiesis. It has yet to be determined if IL-33 also regulates eosinophilopoiesis within the tissue.

Cellular responses to IL-33 have been extensively studied. The ST2 receptor is highly expressed on several “allergy-associated” immune cells, including eosinophils, Th2 cells, ILC2s, mast cells, and basophils, as well as structural cells like epithelial and endothelial cells (31). IL-33 induces the production of type 2-associated cytokines from many of these cell types. Consequently, IL-33 had been presumed to affect eosinophilic inflammation through induction of IL-5—a cytokine known to activate eosinophils. For example, early work concluded that IL-33-induced eosinophilia was dependent on IL-5, but this conclusion was based largely on a neutralizing antibody approach and limited markers for defining eosinophils (32). As outlined in this review, IL-33 is now understood to act directly on eosinophils and regulate their biology, including survival, activation, and adhesion (Figure 1B).

Administration of IL-33 is sufficient to drive *in vivo* eosinophilia in various tissues (5). While IL-33 does not act as an eosinophil chemoattractant (33), several studies show that IL-33 regulates eosinophil survival. For example, transferring ST2 KO eosinophils into recipient mice led to significantly fewer lung eosinophils after allergen challenge than wild-type (WT) eosinophils despite normal migratory functions, implying impaired survival (34). IL-33 also induces GM-CSF that acts in an autocrine fashion to promote survival by inducing the antiapoptotic protein Bcl-x_L_ (35), a response that is negatively regulated by dual-specificity phosphatase 5 (36). Beyond these positive effects of IL-33 on eosinophil survival, IL-33-primed human eosinophils are more susceptible to Siglec-8-induced death; while this priming effect is less effective than IL-5, the two cytokines may function synergistically (37). Thus, the effects of IL-33 on eosinophil survival support a role on both survival and death, most likely in a context-specific fashion.

Interleukin 33 is a potent activator of eosinophils, even more so than IL-5 in terms of triggering degranulation and superoxide release from human eosinophils (38). In mice, IL-33 stimulation alters over 500 genes, many of which are immune related, including IL-6, IL-13, CCL17, CXCL2, CXCL3, and CXCL10 (39). IL-33 can also upregulate several cell-surface markers, including the adhesion molecule CD11b (33), the eotaxin receptor CCR3 (32), and the activation marker CD69 (36).

The functional nature of IL-33-activated eosinophils has been addressed. Transfer of eosinophils activated with GM-CSF, IL-4, and IL-33 into eosinophil-deficient asthmatic mice drove IL-13-dependent mucus production and accumulation of alternatively activated macrophages (40). In a complementary approach, increased IL-13 and alternatively activated macrophages were again observed after intranasal IL-33 administration to ST2 KO mice after adoptive transfer of WT eosinophils; recruitment of several cell types, including macrophages, neutrophils, lymphocytes, and the recipient’s own eosinophils were also observed in this model (32). In the skin, IL-33 has been proposed to directly act on eosinophils to promote fibrosis in an IL-4- and IL-13-dependent manner (41).

### IL-33 on Eosinophilopoiesis

Typically, eosinophils develop in the bone marrow, enter the bloodstream as terminally differentiated cells, and become activated in tissues. However, CD34^+^ progenitors can be detected in blood, and the idea that hematopoiesis can occur in tissues as well as the bone marrow is now accepted. IL-33 appears to have effects on these eosinophil precursors (EoPres) at both locations.

Histologically, eosinophil development was characterized into four classes (I–IV) based on nuclear morphology, granular morphology, and Wright–Giemsa staining. While Class I cells were described as granulocytic but not eosinophilic, Class II cells had small numbers of granules and appeared to have committed to the eosinophil lineage. Prior to terminal differentiation, Class III cells exhibited the characteristic donut-shaped nucleus. Class IV cells were the only eosin-positive cells and maintained the ring-shaped nucleus, which could twist into a figure 8-like structure (42). More recently, EoPres have been phenotyped using flow cytometry for cell-surface markers, including ST2 (Table 1). When the eosinophil lineage-committed progenitor (EoP) was initially identified in mice (43), it was proposed that eosinophils developed in four defined stages within the myeloid pathway. Originating from common myeloid progenitors (CMPs) that differentiate to granulocyte and macrophage progenitors (GMPs), a lineage decision into EoPs then occurs before terminal differentiation into eosinophils. Importantly, although EoP stains with eosin, eosin-negative precursors have been reported (42), suggesting a precursor stage prior to the granulation events occurring in EoP. We identified an EoPre that is eosin negative and exhibits the characteristic donut-shaped nuclei, which is driven by IL-33 exposure (44) (Figure 1A). Both EoP and EoPre are IL-5Rα^+^, but EoPre is Siglec-F^lo^SSC^lo^ whereas EoP is Siglec-F^+^SSC^hi^.

**Table 1:** **Cell-surface markers of cells involved in murine eosinophilopoiesis**.

	Common myeloid progenitor	Granulocyte and macrophage progenitor	Eosinophil precursor (EoPre)	EoP	Mature Eo
Lineage	−	−	high^a^	−	ND
Sca-1	−	−	−	−	−
c-Kit	+	high	−	low	−
CD34	+	+	−	+	−
FcγRII/III	low	high	ND	ND	ND
IL-5Rα	−	−	+	+	+
IL-3R	ND	+	ND	ND	+
IL-4Rα	ND	ND	ND	ND	+
GM-CSFR	ND	+	ND	ND	+
Siglec-F	ND	ND	low	+	+
CCR3	ND	ND	ND	ND	+
Granularity (SSC)	low	low	low	high	high
ST2	+/−	+/−	−	+	+

*+ indicates expression; − indicates no expression; ND indicates expression not determined*.

*^a^Unlike other studies, this study included CD11b in the lineage cocktail and demonstrated that the EoPre is CD11b^hi^*.

From all of the markers defining eosinophils, three appear to be important for defining stages of eosinophil development: in mice, these are IL-5Rα, Siglec-F, and CCR3. IL-5Rα is an indicator of commitment to the eosinophil lineage, as it is a key differentiator between the EoP and earlier stages of development. Siglec-F, originally thought to only mark mature eosinophils and alveolar macrophages outside of the bone marrow, is expressed on EoP; moreover, colony forming assays comparing Lin^−^CD34^+^CD117^int^IL-5Rα^+^ (EoP–IL-5Rα) vs Lin^−^CD34^+^CD117^int^Siglec-F^+^ (EoP–Siglec-F) show that only EoP–IL-5Rα gave rise to pure eosinophils while EoP–Siglec-F cultures generate a mixture of eosinophils and macrophages (45). Thus, Siglec-F appears to mark eosinophil potential in the bone marrow, whereas IL-5Rα indicates commitment to the eosinophil lineage. Finally, CCR3 is a late marker, as it allows eosinophils to enter tissues in response to eotaxin (46).

In humans, the hEoP (IL-5Rα^+^CD34^+^CD38^+^IL-3Rα^+^CD45RA^−^) differentiates directly from the hCMP (Lin^−^CD34^+^CD38^+^IL-3Rα^+^CD45RA^−^IL-5Rα^−^). Furthermore, the hGMP (Lin^−^CD34^+^CD38^+^IL-3Rα^+^CD45RA^+^) is capable of generating neutrophils, monocytes, and basophils (47). Other stages of human eosinophil progenitors have yet to be determined. Although IL-5Rα^+^ progenitors only generate eosinophils, IL-5Rα is expressed in blood on both mature eosinophils and mature basophils (47). Thus, it is unclear if IL-5Rα may be used to identify commitment to the eosinophil lineage as it does in mice. Furthermore, Siglec-8, the human functional paralog of Siglec-F, is expressed at late stages of development of eosinophils, mast cells, and basophils and does not mark eosinophil potential in progenitors the way Siglec-F does in mice (48, 49).

ST2 expression on these progenitors has been controversial. Two studies examining bone marrow stem cells showed opposing results: while Le et al. reported ST2 on Lineage^−^c-Kit^+^Sca-1^+^ cells, CMP, GMP, megakaryocyte-erythroid progenitors (MEP), and common lymphocyte progenitors (50), Mager et al. found no evidence for ST2 on long-term or short-term hematopoietic stem cells, multipotent progenitors (MPP1, MPP2, and MPP3), MEP, CMP, or GMP (51). More recently, Tsuzuki et al. demonstrated that ST2 was expressed on CMP, MEP, and EoP, but not GMP (52). We described a GMP-like cell (Lin^−^Sca1^−^Siglec-F^+^IL-5Rα^−^SSC^lo^c-Kit^hi^CD34^−^) that was ST2^+^, but EoPre was ST2^−^, although this finding came from IL-33-treated mice or *in vitro* cultures (44). These differences in ST2 expression may be partially resolved by new research that redefines the early stages in eosinophil development (53). Using single-cell RNA sequencing of pre-granulocyte macrophage progenitors (Pre-GMP, Lin^−^c-Kit^+^Sca-1^−^CD41^−^CD16/32^−^CD105^−^CD150^−^), Pre-GMP clustered into two groups: GATA-1^+^Flt3^−^ and GATA-1^−^Flt3^+^. By sorting cells from a GATA-1–EGFP reporter and culturing them in eosinophil-promoting conditions, GATA-1^+^ Pre-GMPs generate eosinophils, whereas GATA-1^−^ Pre-GMPs generate neutrophils and monocytes. Drissen et al. proposed that GATA-1^+^-GMPs be renamed eosinophil/mast cell progenitors, and GATA-1^−^ GMPs retain their name. Thus, instead of the classical model (CMP, GMP, EoP, and mature eosinophil), the EoP population can develop independently of the GMP (Figure 1A). This aligns with the description of the hEoP arising from the hCMP and not the hGMP (47). Notably, gene expression of ST2 differentiated the GATA-1^+^ Pre-GMP and GATA-1^−^ Pre-GMP populations (53). Thus, despite continuing debate over ST2 on CMPs and GMPs, eosinophils likely arise from ST2-expressing progenitors.

GATA-1 is a member of the GATA family of transcription factors and known to be critical for eosinophil development. In agreement with the potential of GATA-1^+^ Pre-GMPs to produce eosinophils, human CD34^+^ stem cells transduced to express GATA-1 developed into eosinophils while disruption of GATA-1 expression in mice ablated eosinophils (54). GATA-1 was also one of transcription factors identified as defining the eosinophil lineage through transcriptome analysis comparing GMPs, EoPs, and mature eosinophils; 56 transcription factors were identified including GATA-1, C/EBPε, NFκB, NFAT2, STAT1, STAT3, STAT6, IRF1, IRF2, Helios, and Aiolos (45). However, if and how all of these transcription factors play a role in eosinophil development has yet to be determined, and how IL-33 and ST2 might impact these transcription factors is unclear. ST2 signaling is known to lead to NFκB activation in mature cells (5), and GATA-1 and GATA-2 can regulate ST2 expression through two GATA binding sites upstream of the ST2 promoter (55), supporting a likely interplay at this level.

Several cytokines are important for eosinophil differentiation and maturation from bone marrow. Notably, IL-5 is the hallmark eosinophil-associated cytokine (56). IL-5-overexpressing transgenic mice (NJ.1638) have an excessive number of eosinophils in blood, bone marrow, and tissues with significantly more Class III and Class IV cells in the bone marrow, which, in conjunction with the fact that IL-5Rα marks eosinophil lineage commitment, indicates that IL-5 acts on later stages of development (42). Indeed, IL-5 promotes terminal eosinophil differentiation by upregulating CCR3 (57). IL-5 also upregulates its own receptor on human CD34^+^ cells, but whether this occurs *in vivo* and affects eosinophil development is unclear (58).

The IL-5 receptor shares a β-chain (CD131, CSF2RB) with receptors for IL-3 and GM-CSF. While IL-3 and GM-CSF were initially thought to be important for eosinophil development, we now know that they promote the development of many myeloid cells (56). IL-3 drives mast cell and basophil development and affects mature eosinophils (59). While GM-CSF promotes survival of mature eosinophils, it appears to antagonize eosinophil development *in vitro* (60), although the mechanism has yet to be determined.

Current protocols for developing eosinophils from bone marrow also typically utilize stem cell factor (SCF) and Flt3 ligand (Flt3L) for 3–4 days before IL-5 treatment (61). Flt3L does not seem to be required for eosinophil development (53, 62) while the SCF receptor, c-Kit, is expressed on many stem cells and then lost in the later stages of eosinophil development. Interestingly, we demonstrated that culturing bone marrow cells with SCF and Flt3L for 3 days promoted the expansion of GMP-like cells and mature eosinophils but not the EoPre pool (44).

Recently, we reported that IL-33 may be the missing signal that directs stem cells to commit to the eosinophil lineage. IL-33 treatment significantly expanded the EoPre pool and led to a significant upregulation of IL-5Rα on EoPre, enhancing their responsiveness to IL-5; simultaneously, IL-33 induced IL-5 and mature eosinophil development (44) (Figure 1A). We also demonstrated that NJ.1638 mice had diminished eosinophils in the absence of ST2, indicating that IL-33 regulated the capacity of IL-5 to drive eosinophilia. In agreement with our data, IL-33 treatment of cultured c-Kit^+^ bone marrow cells induced mature eosinophils in an IL-5-dependent manner (32). Interestingly, *in vitro* ST2 KO cells cultured with IL-5 do produce eosinophils, while ST2 KO and IL-33 KO mice have significantly reduced—not absent—eosinophils (44), demonstrating that IL-5-driven eosinophilopoiesis can occur in the absence of IL-33. Unlike GATA-1 that is absolutely required for eosinophil development, the absence of IL-5 may be compensated by other cytokines or factors since IL-5 KO mice also develop basal eosinophil populations (63). Perhaps deleting ST2 and IL-5 or CD131 would be required to ablate eosinophil development.

### IL-33 and Alternative Eosinophilopoiesis Mechanisms within Tissues

There is increasing evidence that progenitors can circulate in the blood and that local hematopoiesis may occur in tissues [reviewed here (64); Figure 1C]. Eosinophil progenitors are increased in the blood and sputum of asthmatic patients (65, 66), but their role in disease not fully understood. Intravenous IL-5 increased not only circulating eosinophil progenitors but also CCR3 expression on CD34^+^ progenitors (67). Similarly, IL-33 increased peripheral blood EoP (52). In response to allergen, CD34^+^CCR3^+^ and Sca-1^+^CCR3^+^ cells proliferated within the lung tissue, demonstrating expansion of local eosinophil lineage-committed stem cells (68). Whether these lung stem cells express ST2 and how IL-33 may affect these cells is unclear. *In vitro*, IL-33 activated EoP to produce chemokines, Th2 cytokines, and pro-inflammatory cytokines, with more IL-9, IL-10, IL-13, IL-1α, IL-1β, IL-6, TNFα, and GM-CSF than mature eosinophils (52); thus, these data implicate EoP as potential regulators over inflammation. Further research is certainly required to determine how eosinophil progenitors contribute to tissue eosinophilia in disease and if IL-33 serves to initiate their responses.

## Conclusion

The biology of IL-33 continues to be a topic of significant discovery and controversy. By focusing on eosinophils, our understanding of this cytokine has begun to be elucidated and shows a complex regulation that extends into homeostasis and disease. While much of this challenges some established views of IL-33 as a local epithelial-derived cytokine, these understandings should significantly impact the interpretations and predictions for using new therapeutics that target this pathway in human health.

## Author Contributions

PB contributed to the overall design and content, as well as edited the final document. LJ contributed to the overall design and content, wrote initial drafts, and designed figures and tables.

## Conflict of Interest Statement

The authors declare that the research was conducted in the absence of any commercial or financial relationships that could be construed as a potential conflict of interest.
